# Retracted: Antioxidant Activity and Characterization of One New Polysaccharide Obtained from* Perigord Truffle* (*Tuber huidongense*)

**DOI:** 10.1155/2018/9686012

**Published:** 2018-01-14

**Authors:** Evidence-Based Complementary and Alternative Medicine

Evidence-Based Complementary and Alternative Medicine has retracted the article titled “Antioxidant Activity and Characterization of One New Polysaccharide Obtained from* Perigord Truffle* (*Tuber huidongense*)” [1] at the request of the authors. The first author says the HPLC and NMR results are not reproducible, while the fourth author did not approve the article's submission and disputes the authorship.
